# Feel What You Say: An Auditory Effect on Somatosensory Perception

**DOI:** 10.1371/journal.pone.0022829

**Published:** 2011-08-08

**Authors:** François Champoux, Douglas M. Shiller, Robert J. Zatorre

**Affiliations:** 1 Centre de recherche interdisciplinaire en réadaptation du Montréal métropolitain/Institut Raymond-Dewar, Montreal, Quebec, Canada; 2 Université de Montréal, École d'orthophonie et d'audiologie, Montreal, Quebec, Canada; 3 Centre de recherche CHU Sainte Justine, Montreal, Quebec, Canada; 4 Centre for Research on Language, Mind and Brain (CRLMB), Montreal, Quebec, Canada; 5 Montreal Neurological Institute, McGill University, Montreal, Quebec, Canada; 6 International Laboratory for Brain, Music and Sound Research (BRAMS), Montreal, Quebec, Canada; Max Planck Institute for Human Cognitive and Brain Sciences, Germany

## Abstract

In the present study, we demonstrate an audiotactile effect in which amplitude modulation of auditory feedback during voiced speech induces a throbbing sensation over the lip and laryngeal regions. Control tasks coupled with the examination of speech acoustic parameters allow us to rule out the possibility that the effect may have been due to cognitive factors or motor compensatory effects. We interpret the effect as reflecting the tight interplay between auditory and tactile modalities during vocal production.

## Introduction

There are some prior demonstrations that changes in sound perception may alter tactile detection by the hands [1]–[3], suggesting that auditory and tactile processing are not always independent. The link between auditory and tactile perception is also supported by recent neuroimaging studies in humans and electrophysiological recordings in animals demonstrating early-stage neural interactions between auditory and somatosensory input [4], although these studies have primarily focused on the influence of tactile input on auditory perception. Far fewer studies have investigated the converse effect (modulation of somatosensory areas following changes in auditory input), with the notable exception of a study by Foxe et al. [5], in which event-related potentials were observed over somatosensory areas following the presentation of auditory pure-tone stimuli.

In the realm of speech, some results suggest that tactile input can influence auditory perception [6]–[9]; for example, it has recently been shown that cutaneous stimuli applied to the neck or the hand influence the perception of voicing in stop consonants [9]. While the specific nature of this striking interaction is uncertain, the converse effect, that is, the influence of auditory stimuli on tactile sensation during speech production or perception, remains unproven. In the present study, we demonstrate a tactile effect that is induced by sound. The audiotactile interaction was discovered unexpectedly during the course of other studies involving the manipulation of acoustical feedback during speech production. Exploratory manipulations appeared to confirm the effect and helped to establish the final experimental design. Pilot studies indicated that the effect was found primarily during the manipulation of acoustic amplitude. Other manipulations of auditory output did not generate a comparable tactile change. The conditions, vocal productions and experimental procedures in the present study were thus chosen to examine the specificity of the effect and rule out the contribution of cognitive or attentional factors or motor compensatory effects [10]–[14]. We present data demonstrating an illusory percept in which amplitude modulation of auditory feedback during voiced speech induces a change in vibrotactile sensation over the lip or the laryngeal regions, an effect that does not occur with frequency modulation or during the production of an unvoiced speech sound. The absence of a tactile effect under conditions in which changes in vibrotactile sensation would not be predicted to naturally co-occur with changes in auditory input indicates that these sensory systems interact in a way that is fine-tuned to the specific nature of auditory stimulus.

## Methods

Thirty-two French-speaking healthy individuals (18 females, 14 males; 21–35 years of age; mean age: 25) participated in the study. All subjects gave their written informed consent to participate in the study, which was performed with approval of the Institutional Review Board of the Faculty of Medicine at McGill University. For all subjects, pure-tone detection thresholds at octave frequencies ranging from 250 to 8000 kHz were within normal limits in both ears. All subjects reported no deficits in tactile perception.

**Figure 1:**
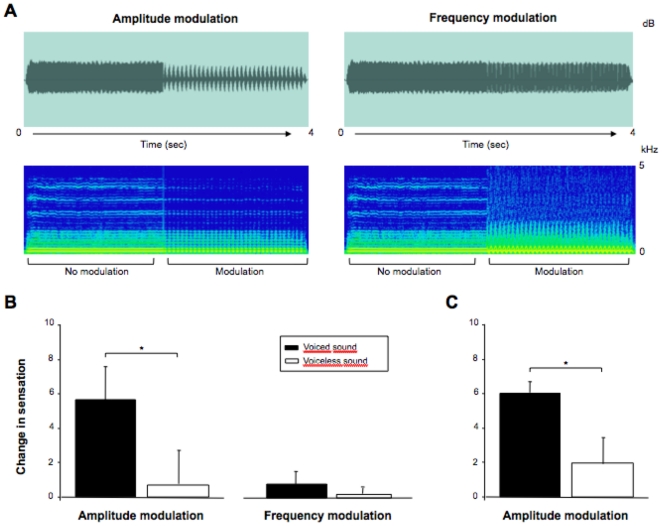
Experimental procedure and mean change in tactile sensation in twenty-two participants. (A) Illustration of the experimental procedure in the active production-listening task. Participants were seated close to a microphone and were repeatedly asked to produce different phonemes for a period of approximately four seconds. The auditory signal from the first two seconds of each phoneme production was not modified whereas the last part of the audio signal was altered in amplitude (left panel) or in frequency (right panel). Top panel shows waveforms (amplitude as a function of time) while bottom panels show spectrograms (frequency as a function of time). (B) Mean change in tactile sensation over the lips region (n = 15) or (C), the throat/larynx region (n = 7) and standard deviations while producing the voiced (black bar) and the voiceless sound (white bar). * : *p*<.005.

In Experiment 1, fifteen participants were seated close to a microphone and were asked to repeatedly produce the sustained vowel /u/ (as in “b**oo**t”) or the fricative /∫/ (as in “bu**sh**”) for a period of approximately four seconds and at an inter-stimulus interval of 5–10 seconds. The subject listened to the sound of his or her own voice through insert earphones. In order to avoid any direct somatosensory stimulation to ear canal, the intensity of the sound production was kept at a relatively low level (55–60 dB HL) throughout the testing session (monitored by the experimenter with the help of a VU-meter). Following two seconds of unaltered auditory feedback at the beginning of each trial, the audio signal was altered in real-time (see Fig. 1a) using an amplitude (loudness) modulation or a frequency (pitch) modulation effect (Logic Pro software, Apple, USA). During the two-second portion of altered auditory feedback, amplitude or frequency modulation was introduced at a rate of 16 Hz, with amplitude ranging from 0–100% and frequency ranging from −0.5 to +0.5 octave (Figure 1A). Note that the one-octave variation in frequency is considerably greater than the threshold for detection of frequency-modulated complex acoustic signals [15], and is comparable to the large variations in fundamental and formant frequency associated with speech production in some contexts, such as infant-directed speech [16]. Following each phoneme production, participants were immediately asked to quantify any change in perceived tactile sensation over the lip region following the onset of the feedback modulation on a scale of 1 (no change in pulsation/vibration) to 10 (strong change in pulsation/vibration).

Two other tasks, using the same procedure but different auditory and tactile stimulations, were administrated in order to examine the specificity of the effect. In order to determine if a change in tactile sensation would be observed during the production of a different class of phoneme (e.g., fricatives) and if the effect was specific to one tactile region, a second experiment was carried out. Seven additional participants (four males) produced the voiced fricative /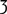/ (as in “plea**s**ure”) or the unvoiced fricative /∫/ (as in “bu**sh**”). For these sounds, auditory feedback was modulated in amplitude only. Participants were asked to focus on the tactile sensation over the laryngeal region (i.e. the throat) and report any change during the course of sound production.

In order to rule out the possibility that the effect may have been due to cognitive factors, such as a shift in the response criterion associated with the different auditory manipulation types (i.e., a bias toward the amplitude modulation), a third experiment was carried out exploring perceived changes in vibrotactile sensation at the hand during the passive listening of amplitude and frequency-modulated auditory stimuli. Under these conditions, ten participants (four males; all right-handed) listened to either a periodic (sawtooth) or aperiodic sound (speech-shaped noise) at 60 dB for a duration of four seconds. Simultaneous with the auditory stimulation, a suprathreshold vibrotactile stimulus (100 Hz; 0.1 mm displ.) was applied to right hand using a vibratory stimulator (Silent Call Vibrator, Silent Call communications, Michigan). After 2-seconds of auditory-tactile stimulation, the auditory stimulus was modulated in precisely the same manner as in Experiment 1∶16 Hz modulation of frequency (+/−0.5 octave) or amplitude (0–100%). Subjects were asked to report the degree of perceived change in vibratory sensation at the hand on a scale of 1 (no change) to 10 (large change). In the three experimental tasks, each condition was presented 10 times each in a pseudorandom order.

## Results

When a repetitive variation in the sound's amplitude was introduced during vowel production, all participants systematically reported an increase in vibrotactile sensation (mean rating = 5.76±1.95; see Figure 1B). In contrast, frequency modulation had only a negligible effect on tactile perception during vowel production (mean rating: 0.87±1.91). The perceived change in tactile sensation was also nearly zero in all participants during the fricative production, whether the sounds were modulated in frequency (mean rating: 0.24±0.16) or in amplitude (mean rating: 0.89±0.41). A 2×2 ANOVA with modulation type (amplitude, frequency) and phoneme (vowel, fricative) as factors was conducted. There was a reliable main effect of modulation type (*F* = 50.15, *p*<.001), and the interaction between factors was significant (*F* = 53.53, *p*<.001). Post-hoc analysis revealed a significant difference between the two phoneme conditions during amplitude modulation (*t* = 8.85; *p*<.001), but not during frequency modulation (*t* = 1.73; *p* = .106). The difference between amplitude and frequency modulation was also reliable during vowel production (*t* = 8.12; *p*<.001), but not during fricative production (*t* = 1.95; *p* = .071).

As in Experiment 1, the manipulation of auditory feedback was found to alter laryngeal sensation in the second experiment (Figure 1C); tactile sensations were significantly greater during the voiced sound compare to the unvoiced sound condition (*t* = −4.5, *p* = .004). In order to rule out the possibility that the observed changes in vibrotactile sensation during vowel production were due to a change in speech output resulting from the feedback manipulation (i.e., a motor compensatory response), an analysis of the acoustical output was carried out. For each trial, measures of vocal intensity (loudness), fundamental frequency (pitch), and the first two formants (vocal tract resonant frequencies; F1 and F2) were obtained during a 0.5 sec window prior to and subsequent to the onset of the feedback shift (with the center of the analysis windows offset by +/−0.75 seconds from the beginning of the feedback shift). The pre-post difference in acoustic parameters was examined for both the amplitude modulation condition (in which an increase in vibrotactile sensation was found), and the frequency modulation condition (in which no change in tactile sensation was observed). The magnitude of the changes were in all cases comparable between the amplitude and frequency modulation conditions and no reliable difference between modulation types was observed (pitch: *t* = −1.43, *p* = .20; amplitude: *t* = −.68, *p* = .52; F1: *t* = −1.56, *p* = .16; F2: *t* = −0.81, *p* = .45).

In the passive-listening task (third task), however, very little change in tactile sensation was reported under conditions of both frequency and amplitude modulation of auditory input (mean rating below 1.5 for all conditions), hence no reliable differences between the two conditions were found (*p*>.05).

## Discussion

The data reported here demonstrate that the alteration of auditory feedback can produce a change in tactile sensation, but only under specific combinations of sound production and modulation conditions. Listeners consistently reported a change in vibrotactile sensation in their lips or their throat under conditions of amplitude modulation during the production of vocalized speech, whereas no such changes were reported under conditions of frequency modulation, or during the production of an unvoiced fricative. Importantly, while the perceived vibrotactile sensation only arose during the production of vocalized speech, the change in sensation induced by the manipulation of auditory feedback did not result from a change in voicing properties. The physical properties of the acoustic signal being produced by the subject were found to be comparable under amplitude and frequency modulation conditions, only one of which (amplitude modulation) resulted in the vibrotactile effect. This novel auditory-tactile effect reveals a tight coupling between orosensory and auditory sensory processing precisely under those conditions in which both sensory systems would experience simultaneous changes in stimulation during speech production: *i)* during the production of voiced sounds, which unlike the production of unvoiced fricatives, is associated with oscillatory patterns of intra-oral air pressure (typically in the range of 100–200 Hz) detectable by mechanoreceptors in the oral tissues, and *ii)* during large changes in loudness (e.g., 0–100%), which unlike the modulation of frequency, is linked with large changes in the magnitude of intraoral pressure (and its associated vibrotactile sensation).

A number of motor compensatory effects have been reported in response to perceived changes in auditory feedback during vocalized speech production, raising the possibility that such behavioral changes might underlie the sensory changes observed in the present study. These motor responses include auditory-labial reflexes [12], compensatory adjustments of speech amplitude in response to perceived changes in vocal loudness [13] and laryngeal (pitch-altering) responses that occur when the fundamental frequency of vowels is unexpectedly altered during speech production and singing [14]–[16]. While none of these prior studies have demonstrated motor responses to rapid (e.g., 16 Hz) modulation of amplitude or frequency, an examination of speech acoustic parameters was nonetheless carried out in the present study to rule out the possible contribution of such motor factors to the observed perceptual changes. Small fluctuations in pitch, loudness and vocal tract resonant properties were observed between the period preceding and following the onset of the feedback manipulation, as would be expected during the course of any sustained vowel production. Critically, no difference in these acoustic parameters was found between the frequency and amplitude modulated conditions, indicating that such changes in speech output were not responsible for the sensory outcome (which was reported only under conditions of amplitude modulation).

One could also argue that directing attention to a region of the skin involved in speech production (the lips or the throat) would bias responses on the subjective response scale we used. However, we observed a specific link between tactile sensation and amplitude modulation (not frequency modulation), and only during the production (not during passive-listening) of a vowel (not a voiceless fricative). These dissociations indicate that the effect is not related to task demand characteristics or simply heightened attention to tactile sensations at the lips or the throat. The present demonstration, however, does not rule out the possible integration of auditory and tactile processing in other orofacial regions.

In light of the present findings, two important questions can be raised, both of which are relevant to the interpretation of the current results: *i*) Why does one observe an impact of auditory input on the perception of vibrotactile stimulation at the lips or the throat only under certain conditions? And *ii*) What does the result say about the role of somatosensory input in the control of articulator motion during speech production? Our interpretation of the result is straightforward: One observes a link between the two modalities precisely under those conditions in which both would be expected to experience simultaneous stimulation during speech production. There is a physical explanation for this. The large 100–200 Hz oscillations in air pressure resulting from voiced speech are likely to be detectable by both the ear (as sound) and the mechanoreceptors in the orofacial skin (as a vibrotactile sensation), owing to the heightened sensitivity of both systems within that frequency range [17]. In contrast, during the production of the voiceless fricative, air pressure variation is not periodic but random in nature (owing to the sound source being a constriction of the air channel, rather than the vibration of the vocal folds), with most of the energy at much higher frequencies (>3000 Hz). Furthermore, during the production of sibilant fricative /∫/, the air stream is partially deflected off of the upper incisors, thus reducing the direct flow of air over the labial surface. As a consequence, while the fricative is highly salient acoustically, the occurrence of vibration at the lips or the throat would be minimal. A similar mechanism underlies the effect of amplitude modulation on perceived vibrotactile sensation. Amplitude modulation (i.e., louder and softer speech) corresponds with changes in the magnitude of air-pressure variation. Since air pressure variation is the common underlying physical stimulus for both auditory and vibrotactile modalities during speech production, changes in amplitude would alter the strength of the vibrotactile percept at the same time as changes in loudness would be perceived auditorily. In contrast, frequency modulation of the acoustic signal results in no change in the magnitude of the air pressure change. Hence, modulation of frequency would not be expected to coincide with changes in the strength of the vibrotactile percept.

As for the possible role of vibrotactile input in the control of speech production, given the observed link between auditory and tactile input during amplitude modulation, it is possible that the vibrotactile input at the lips or the throat, in parallel with the auditory system, may provide the nervous system with information related to the amplitude (i.e., loudness) of vocalized sound production. The loudness of the speech signal arises from an interaction between a number of physiological systems working in concert, including respiratory, laryngeal and oral mechanisms. A change in any of these systems could result in a change in acoustic amplitude. The precise control of loudness is certainly possible in the absence of auditory input. While there are a number of possible sources of somatosensory feedback about amplitude (e.g., proprioceptive input from respiratory and laryngeal muscles, or vibrotactile input from the tissues of the larynx), the lips, which lie at the output of the entire system, may provide particularly valuable information about the net impact of all systems working together.

Combined with previous findings, our results support the possible involvement of somatosensory input in speech production [18], [19] and extend it to show that just as somatosensory input can alter auditory percepts, so can auditory inputs alter somatosensory percepts. The results of the present study are complementary to the study of Gick and Derrick [9] by demonstrating that the auditory-tactile relationship during speech production is a two-way relationship - something never clearly demonstrated before this paper. We interpret the present audiotactile effect as reflecting the tight interplay between auditory and tactile modalities during vocal production, though the precise role of labial tactile sensation in the control of vowel production remains unknown. Future electrophysiological or neuroimaging investigations may confirm the origin of this phenomenon and its possible importance in motor and language learning.
